# Indirect Effect of Pneumococcal Conjugate Vaccines on Pneumococcal Colonization: Persistence and Dynamics of Vaccine Serotypes in Sicily (Italy) 11 Years Postintroduction, 2009–2020

**DOI:** 10.1093/infdis/jiag150

**Published:** 2026-03-07

**Authors:** Fabio Tramuto, Giulia Randazzo, Arianna Santino, Giuseppe Sferlazza, Adriana Previti, Giorgio Graziano, Claudio Costantino, Walter Mazzucco, Emanuele Amodio, Francesco Vitale, Carmelo Massimo Maida

**Affiliations:** Department of Health Promotion, Mother and Child Care, Internal Medicine and Medical Specialties “G. D’Alessandro”—Hygiene Section, University of Palermo, Palermo, Italy; Regional Reference Laboratory for Molecular Surveillance of Influenza, Clinical Epidemiology Unit, University Hospital “Paolo Giaccone”, Palermo, Italy; Regional Reference Laboratory for Molecular Surveillance of Influenza, Clinical Epidemiology Unit, University Hospital “Paolo Giaccone”, Palermo, Italy; Regional Reference Laboratory for Molecular Surveillance of Influenza, Clinical Epidemiology Unit, University Hospital “Paolo Giaccone”, Palermo, Italy; Regional Reference Laboratory for Molecular Surveillance of Influenza, Clinical Epidemiology Unit, University Hospital “Paolo Giaccone”, Palermo, Italy; Regional Reference Laboratory for Molecular Surveillance of Influenza, Clinical Epidemiology Unit, University Hospital “Paolo Giaccone”, Palermo, Italy; Regional Reference Laboratory for Molecular Surveillance of Influenza, Clinical Epidemiology Unit, University Hospital “Paolo Giaccone”, Palermo, Italy; Department of Health Promotion, Mother and Child Care, Internal Medicine and Medical Specialties “G. D’Alessandro”—Hygiene Section, University of Palermo, Palermo, Italy; Regional Reference Laboratory for Molecular Surveillance of Influenza, Clinical Epidemiology Unit, University Hospital “Paolo Giaccone”, Palermo, Italy; Department of Health Promotion, Mother and Child Care, Internal Medicine and Medical Specialties “G. D’Alessandro”—Hygiene Section, University of Palermo, Palermo, Italy; Regional Reference Laboratory for Molecular Surveillance of Influenza, Clinical Epidemiology Unit, University Hospital “Paolo Giaccone”, Palermo, Italy; Department of Health Promotion, Mother and Child Care, Internal Medicine and Medical Specialties “G. D’Alessandro”—Hygiene Section, University of Palermo, Palermo, Italy; Department of Health Promotion, Mother and Child Care, Internal Medicine and Medical Specialties “G. D’Alessandro”—Hygiene Section, University of Palermo, Palermo, Italy; Regional Reference Laboratory for Molecular Surveillance of Influenza, Clinical Epidemiology Unit, University Hospital “Paolo Giaccone”, Palermo, Italy; Department of Health Promotion, Mother and Child Care, Internal Medicine and Medical Specialties “G. D’Alessandro”—Hygiene Section, University of Palermo, Palermo, Italy; Regional Reference Laboratory for Molecular Surveillance of Influenza, Clinical Epidemiology Unit, University Hospital “Paolo Giaccone”, Palermo, Italy

**Keywords:** *Streptococcus pneumoniae*, colonization, serotype, vaccine, Italy

## Abstract

**Background:**

In Italy, evidence on the long-term effects of pneumococcal conjugate vaccines on nasopharyngeal carriage is still limited. This study assessed pneumococcal carriage prevalence, serotype distribution, and temporal trends during the decade after 13-valent pneumococcal conjugate vaccine (PCV13) introduction and before the severe acute respiratory syndrome coronavirus 2 (COVID-19) pandemic.

**Methods:**

Oropharyngeal samples were collected from 12 733 individuals of all ages presenting with influenza-like illness within the national respiratory pathogens surveillance network. Streptococcus pneumoniae detection and serotyping were performed using real-time PCR-based assays.

**Results:**

Overall pneumococcal carriage was 27.1%. The highest prevalence occurred in children aged 2–4 years (51.6%), while colonization was about 10% among adults, including those ≥75 years. After vaccine introduction, PCV serotypes declined markedly, accompanied by increased nonvaccine serotypes. Following years of sustained pediatric vaccination, vaccine serotypes re-emerged, replacing previously expanding non-PCV types. Some vaccine serotypes associated with higher invasive disease risk persisted despite high vaccination coverage. Serotype distribution differed significantly by age, and viral coinfection–especially hRSV–appeared to increase pneumococcal colonization likelihood.

**Conclusions:**

Pneumococcal carriage remained common across all ages despite long-standing pediatric vaccination, with continued circulation of both vaccine and nonvaccine serotypes. Viral coinfection may facilitate colonization, highlighting the need for ongoing surveillance and adaptive vaccination strategies.

## Teaser

From 2009 to 2020, pneumococcal carriage remained substantial across all ages despite sustained pediatric vaccination. Vaccine serotypes initially declined but later re-emerged, replacing previously expanding non-PCV types. Persistent PCV serotypes underscore evolving pneumococcal dynamics and the need for continued surveillance.

Infections caused by *Streptococcus pneumoniae* (pneumococcus) continue to represent a major global cause of illness and death. This microorganism is responsible for a wide spectrum of clinical conditions, including severe manifestations collectively referred to as invasive pneumococcal disease (IPD) [1], as well as more frequent community-acquired infections, such as otitis media, sinusitis, conjunctivitis, and pneumonia. Invasive pneumococcal disease is characterized by the detection of pneumococcus in normally sterile body sites, such as blood or cerebrospinal fluid, and is associated with a substantial burden of complications and high fatality rates.

The highest burden of IPD is among infants, the elderly, and immune-compromised patients, although younger adults are also at risk [2]. A key determinant of pneumococcal virulence is the polysaccharide capsule, which enables the pathogen to evade host immune responses. More than 100 immunologically distinct capsular serotypes have been identified, each differing in invasive potential and prevalence. Since currently licensed vaccines rely on the induction of serotype-specific antibodies targeting the capsular polysaccharides, they provide protection against the serotypes included in their formulation. Nevertheless, similarities in composition and structure of some polysaccharide antigens may result in cross-reactivity in immunized individuals against closely related serotypes [3]. Most commonly, vaccine-induced cross-reactive antibodies occur between those serotypes that are categorized in the same serogroup [4], as demonstrated within the serogroups 6, 9, 15, 19, 20, and 23 [5].

Extensive evidence demonstrates that the introduction of large-scale childhood vaccination programs has led to a marked decline in pneumococcal disease in many countries. The benefits extend beyond vaccinated children, also providing indirect protection of nonvaccinated subjects linked to reduced pathogen transmission within a community [6]; nevertheless, some serotypes contained in pediatric pneumococcal conjugate vaccines (PCVs) have remained a persistent cause of disease among older adults [7]. In addition, following the resolution of pandemic-related restrictions associated with COVID-19, the burden of IPD increased again in several nations, including Italy. According to the most recent national surveillance data, the overall incidence of pneumococcal IPD in Italy rose from 0.84 cases per 100 000 inhabitants in 2021 to 3.02 per 100 000 in 2023, with the highest rates observed in children under 1 year of age (10.41 per 100 000) and adults over 65 years (7.45 per 100 000) [8]. Across Europe, estimates of IPD incidence range between 0.1 and 12.2 cases per 100 000 population [9], although the true incidence remains uncertain due to variability in reporting practices, diagnostic methods, and surveillance structures [10].

Sicily was the first Italian region to implement universal pneumococcal vaccination for infants within its regional immunization plan. In 2004, the 7-valent pneumococcal conjugate vaccine (PCV7) was introduced using a 2 + 1 schedule in infants aged 2 months or older and was replaced by PCV13 in 2010. Additionally, the 23-valent pneumococcal polysaccharide vaccine (PPSV23) was recommended for adults aged ≥65 years and for those aged ≥19 years with chronic conditions, to be administered sequentially after PCV13. Since 2023, this recommendation has shifted to a PCV20 + PPSV23 sequential schedule for individuals ≥60 years and for adults ≥19 years with clinically relevant comorbidities. In Sicily, vaccination coverage among newborns and toddlers has consistently exceeded 90% in birth cohorts from 2010 to 2020 [11], while uptake among adults and the elderly remains substantially lower [12].

Beyond clinical infections, *S. pneumoniae* frequently colonizes the nasopharynx of healthy individuals, particularly children, who serve as the main reservoir facilitating bacterial transmission. Colonization is recognized as a critical precursor to both disease development and community spread [13], although the mechanisms governing the transition from carriage to invasive disease are not fully understood.

One well-documented consequence of pediatric PCV vaccination is a marked reduction of vaccine serotypes (VTs) in carriage and disease, accompanied by an expansion of nonvaccine serotypes (NVTs), a process known as serotype replacement, observed both in vaccinated and unvaccinated populations [14–16]

In this context, surveillance of pneumococcal carriage represents an important tool to assess selective vaccine pressure and, therefore, to guide the adoption of higher-valent vaccines, such as PCV15, PCV20, and PCV21 [17]. However, most carriage studies have been limited to short- or medium-term observation periods [18] or have focused at pediatric populations [19], making them inadequate for evaluating the long-term effects of vaccination on both carriage prevalence and serotype distribution, especially in older age groups.

The present observational study aims to retrospectively evaluate pneumococcal carriage and serotype-specific patterns across different age groups in Sicily, an Italian region characterized by high pediatric PCV coverage, over a decade following the introduction of universal childhood vaccination and preceding the onset of the COVID-19 pandemic.

## METHODS

### Study Design and Specimen Collection

A cross-sectional observational study was conducted to determine the prevalence, serotype composition, and temporal patterns of oropharyngeal colonization by *S. pneumoniae* among residents of Sicily, the fifth most populated region of Italy. To achieve this objective, oropharyngeal samples were obtained with the support of general practitioners and pediatricians participating in RespiVirNet, the Italian surveillance network monitoring influenza-like illnesses (ILIs) in Sicily [20]. Sampling took place during winter from 2009 until late January 2020, immediately preceding the onset of the COVID-19 public health emergency. Oropharyngeal specimens were collected using standardized flocked swabs and processed at the Sicilian Regional Reference Laboratory located at the “P. Giaccone” University Hospital in Palermo.

### Laboratory Procedures

All respiratory samples were subjected to molecular screening to detect influenza viruses, respiratory syncytial virus (hRSV), and pneumococcus. Detection of influenza and hRSV was carried out using real-time PCR (RT-PCR) protocols previously published in the literature [21, 22], whereas pneumococcal carriage was identified through a specific RT-PCR assay targeting the pneumococcal autolysin gene (lytA). Samples testing positive for *S. pneumoniae* (lytA-positive) were subsequently serotyped using a panel of single-plex RT-PCR assays aimed at detecting serotypes included in the PPSV23. Because of the genetic similarity of capsular loci within defined serogroups, some assays were limited to serogroup-level identification, such as 6A/6B, 7A/7F, 9L/9N, 9A/9 V, 11A/11D/11E, 12F/44, 18B/18C, 19B/19F, 22A/22F, and 33A/33F/37. All tests were performed in duplicate, incorporating both negative and plasmid-positive controls, following the Centers for Disease Control and Prevention (CDC) recommendations [23] with minor laboratory modifications. An RT-PCR result was considered negative when no amplification occurred after 40 cycles.

### Statistical Analysis

Pneumococcal serotypes were classified into 4 categories: “PCV7 serotypes,” “additional PCV13 serotypes,” “non-PCV13 PPSV23 serotypes,” and “NVTs.” Participants were grouped into 10 age classes: ≤12 months, 13–23 months, 2–4 years, 5–9 years, 10–14 years, 15–24 years, 25–44 years, 45–64 years, 65–74 years, and ≥75 years. Descriptive statistics (frequency, percentage, median, and interquartile range) were used to summarize demographic and clinical characteristics. Associations between pneumococcal carriage and participant factors were evaluated using logistic regression, with odds ratios (ORs/adjOR) and 95% CIs. Age and sex were included as a priori variables, and stratified analyses were conducted to assess effect modification. Statistical significance was defined as *P* ≤ .05 (2-tailed). Data were analyzed using STATA MP v19.5 (StataCorp, College Station, TX, United States).

### Ethical Considerations

The study followed Italian data protection laws and the principles of the Declaration of Helsinki. Written informed consent was obtained from all participants or from parents/legal guardians of minors. All authors had full access to the data and took responsibility for the publication.

## RESULTS

### Study Population

A total of 12 733 individuals were included (Supplementary Figure 1) and analyzed according to their pneumococcal colonization status. Their demographic and clinical characteristics are summarized in Table 1. The overall male-to-female ratio (M:F & 1.10) remained consistent throughout the surveillance period, ranging from 0.95 to 1.50 across individual seasons (data not shown). The median age of enrolled subjects was 13 years (interquartile range [IQR] & 43), and more than half of the participants (approximately 52%) were children or adolescents aged ≤14 years, although all age groups were represented. With the exception of the 2009–2010 surveillance season, heavily influenced by the influenza A(H1N1)pdm09 pandemic, most participants were recruited from the community (77.5%, n & 8240/10 625), rather than from hospital settings.

**Table 1.** Demographic Characteristics of the Study Population According to Pneumococcus Detection

				Logistic Regression		
		lytA-neg (%)	lytA-pos (%)	adjOR^a^	95% CI	*P*
Study population (n [%])	n = 12 733	9281 (72.9)	3452 (27.1)			
Sex (n [%])						
Male	6686 (52.5)	4865 (72.8)	1821 (27.2)	REF		
Female	6047 (47.5)	4416 (73.0)	1631 (27.0)	1.01	0.94–1.10	.738
Age (years; median [IQR])	13 (43)	24 (45)	5 (9)			
Age groups (years) (n [%])	n = 12 604					
≤12 m	448 (3.6)	279 (62.3)	169 (37.7)	6.52	4.70–9.05	<.001
13–23 m	595 (4.7)	331 (55.6)	264 (44.4)	8.58	6.28–11.73	<.001
2–4	1869 (14.8)	904 (48.4)	965 (51.6)	11.49	8.67–15.23	<.001
5–9	2359 (18.7)	1406 (59.6)	953 (40.4)	7.29	5.52–9.64	<.001
10–14	1290 (10.2)	927 (71.9)	363 (28.1)	4.21	3.14–5.65	<.001
15–24	874 (6.9)	763 (87.3)	111 (12.7)	1.57	1.12–2.18	.008
25–44	1749 (13.9)	1530 (87.5)	219 (12.5)	1.54	1.14–2.08	.005
45–64	2023 (16.1)	1772 (87.6)	251 (12.4)	1.52	1.13–2.05	.006
65–74	703 (5.6)	621 (88.3)	82 (11.7)	1.42	0.99–2.02	.051
≥75	694 (5.5)	635 (91.5)	59 (8.5)	REF		
Healthcare settings (n [%])^b^	n = 10 625					
Community	8240 (77.5)	5174 (62.8)	3066 (37.2)	2.78	2.36–3.26	<.001
Hospital	2387 (22.5)	2166 (90.8)	219 (9.2)	REF		

^a^aOR: OR adjusted by age.

^b^Excluding the surveillance season 2009–2010.

### Oropharyngeal Carriage of *Streptococcus pneumoniae*

Overall, 27.1% of the population (n = 3452/12 733) tested positive for the pneumococcal lytA gene and were therefore classified as carriers. Carriage prevalence was similar between males and females (Table 1). However, the age distribution differed significantly between carriers and noncarriers: pneumococcal carriers had a median age of 5 years, compared with 24 years among noncolonized individuals. Colonization was markedly higher among children, particularly those ≤14 years, where prevalence ranged from 28.1% to 51.6%. The highest risk of carriage was observed in children aged 2–4 years (OR = 11.49; 95% CI: 8.67–15.23). In contrast, pneumococcal carriage was relatively uncommon in adults aged ≥45 years, where prevalence varied between 8.5% and 12.4% (Table 1; Supplementary Figure 2).

The majority of carriers were identified in the community, and individuals outside hospitals were nearly 3 times more likely to carry pneumococcus than hospitalized subjects (adjOR = 2.78; 95% CI: 2.36–3.26). Colonization also demonstrated a strong seasonal pattern, peaking during winter months. Carriage rates increased by approximately 5–6-fold in December through February, compared to September, taken as the reference month (Supplementary Figure 3).

Because the study was conducted among individuals presenting with ILI, the role of coinfection with influenza viruses or respiratory syncytial virus was assessed (Table 2). Influenza virus alone was detected in 45.1% (n & 3590/7968) of tested individuals, predominantly type A (70.9%, n & 2547/3590). In contrast, hRSV alone was detected in 12.3% (n & 614/4992) of subjects, with subgroup A and B almost evenly distributed (46.4% vs 53.6%). Both influenza and hRSV were positively associated with pneumococcal carriage, but the strength of association was greater for hRSV (influenza adjOR & 1.19; 95% CI: 1.07–1.31; hRSV adjOR & 1.97; 95% CI: 1.64–2.36).

**Table 2.** Pneumococcal Colonization and Codetection of Influenza Virus or hRSV

				Logistic Regression		
		lytA-neg (%)	lytA-pos (%)	adjOR^a^	95% CI	*P*
Influenza infection (n [%])^b^	n = 7968					
No	4378 (54.9)	3139 (71.7)	1239 (28.3)	REF		
Yes	3590 (45.1)	2318 (64.6)	1272 (35.4)	1.19	1.07–1.31	.001
Influenza type A	2547 (70.9)	1647 (71.1)	900 (70.8)			
Influenza type B	1043 (29.1)	671 (28.9)	372 (29.2)			
hRSV infection (n [%])^c^	n = 4992					
No	4378 (87.7)	3139 (71.7)	1239 (28.3)	REF		
Yes	614 (12.3)	292 (47.6)	322 (52.4)	1.97	1.64–2.36	<.001
hRSV-A	285 (46.4)	123 (42.1)	162 (50.3)			
hRSV-B	329 (53.6)	169 (57.9)	160 (49.7)			

Abbreviation: hRSV, respiratory syncytial virus.

^a^aOR: OR adjusted by age.

^b^Subjects negative for hRSV.

^c^Subjects negative for influenza virus.

**Figure 1.**
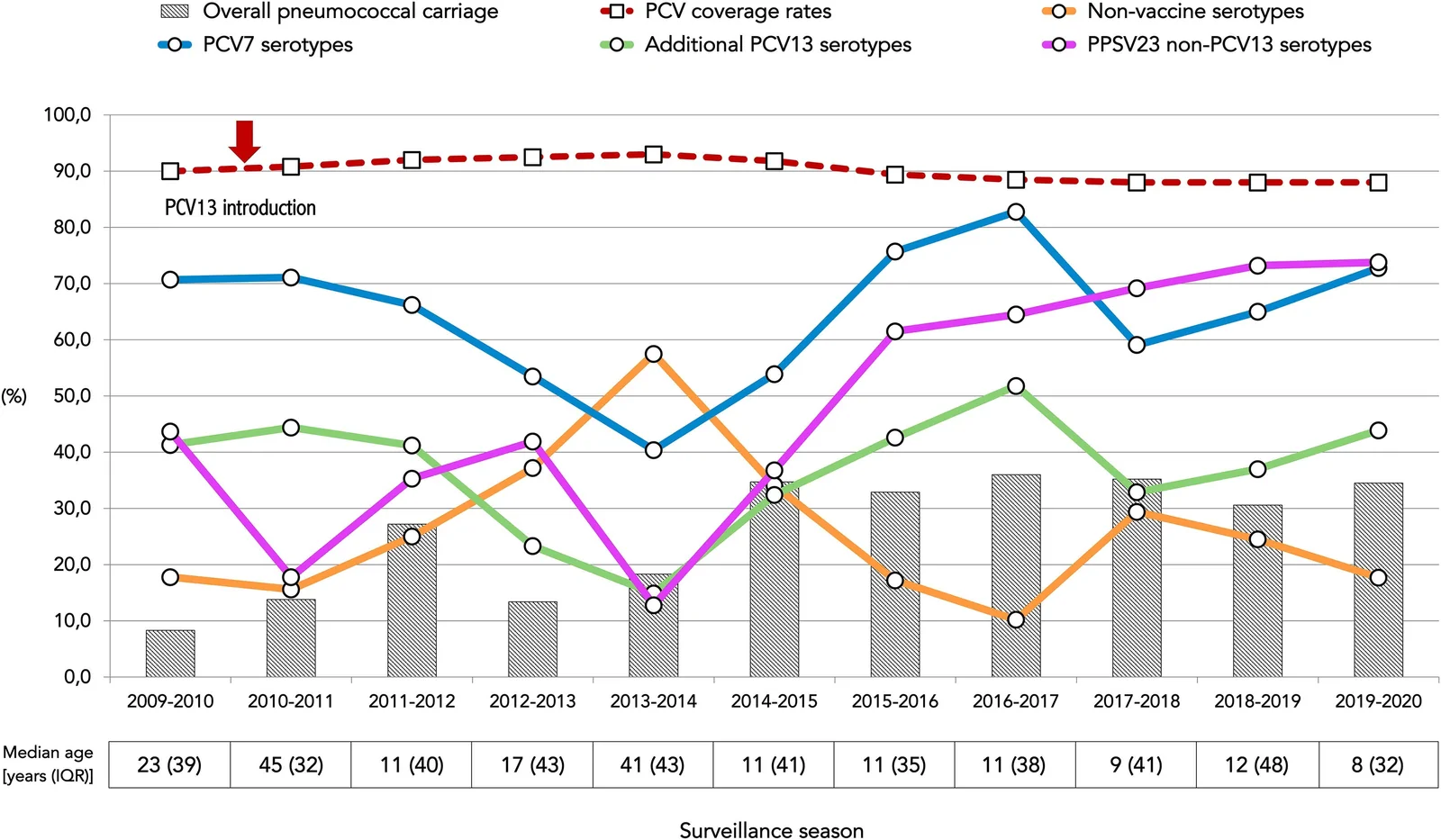
Prevalence of pneumococcal carriage, PCV coverage rates and frequency of pneumococcal serotypes according to different vaccine sets, by surveillance season. PCV, pneumococcal conjugate vaccine.

Fluctuations in pneumococcal carriage prevalence across surveillance seasons likely reflected year-to-year changes in population age structure. Figure 1 also overlays pneumococcal carriage trends with vaccine coverage in Sicily following the introduction of PCV13 in mid-2010. Notably, PCV7-/PCV13-type serotypes steadily decreased during the first 5 surveillance seasons, reaching a minimum in 2013–2014, followed by a gradual increase. A similar pattern was observed among additional PPSV23 serotypes. In contrast, the proportion of NVTs peaked in 2013–2014 before declining in subsequent years.

### Serotype-Specific Carriage

**Figure 2.**
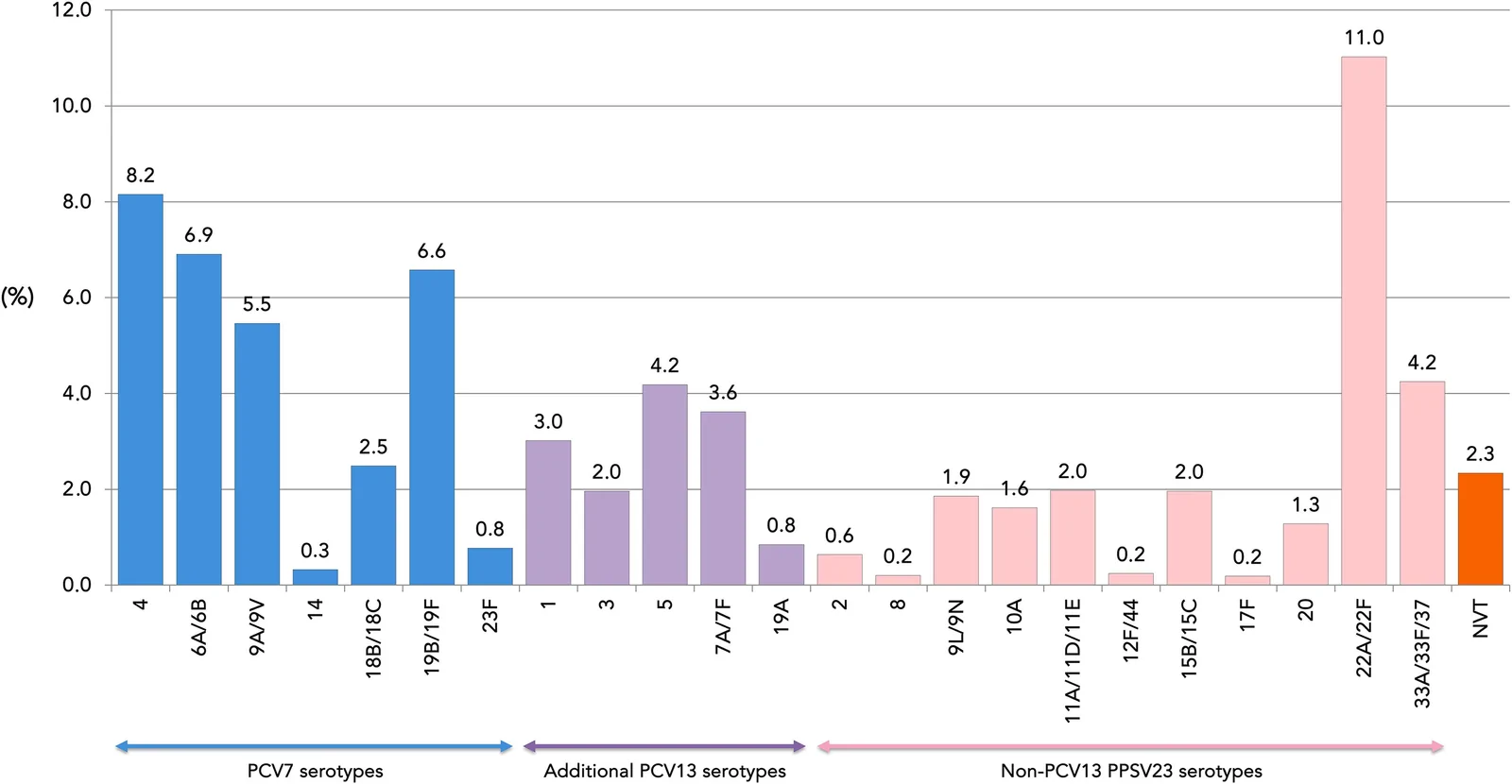
Prevalence of *Streptococcus pneumoniae* serotypes/serogroups included in PCV7, PCV13, or PPSV23 vaccines. Pooled surveillance seasons. NVTs, nonvaccine serotypes; PCV7, 7-valent pneumococcal conjugate vaccine; PCV13, 13-valent pneumococcal conjugate vaccine; PPSV23, 23-valent pneumococcal polysaccharide vaccine.

Serotype distribution across the entire surveillance period is shown in Figure 2. The 5 most frequently detected serotypes/serogroups were 22A/22F, 4, 6A/6B, 19B/19F, and 9A/9V, each with a prevalence ≥ 5.5%. Four of these were included in PCVs. All other detected serotypes/serogroups were less common, and 2.3% of carriers harbored serotypes absent from current vaccine formulations.

**Figure 3.**
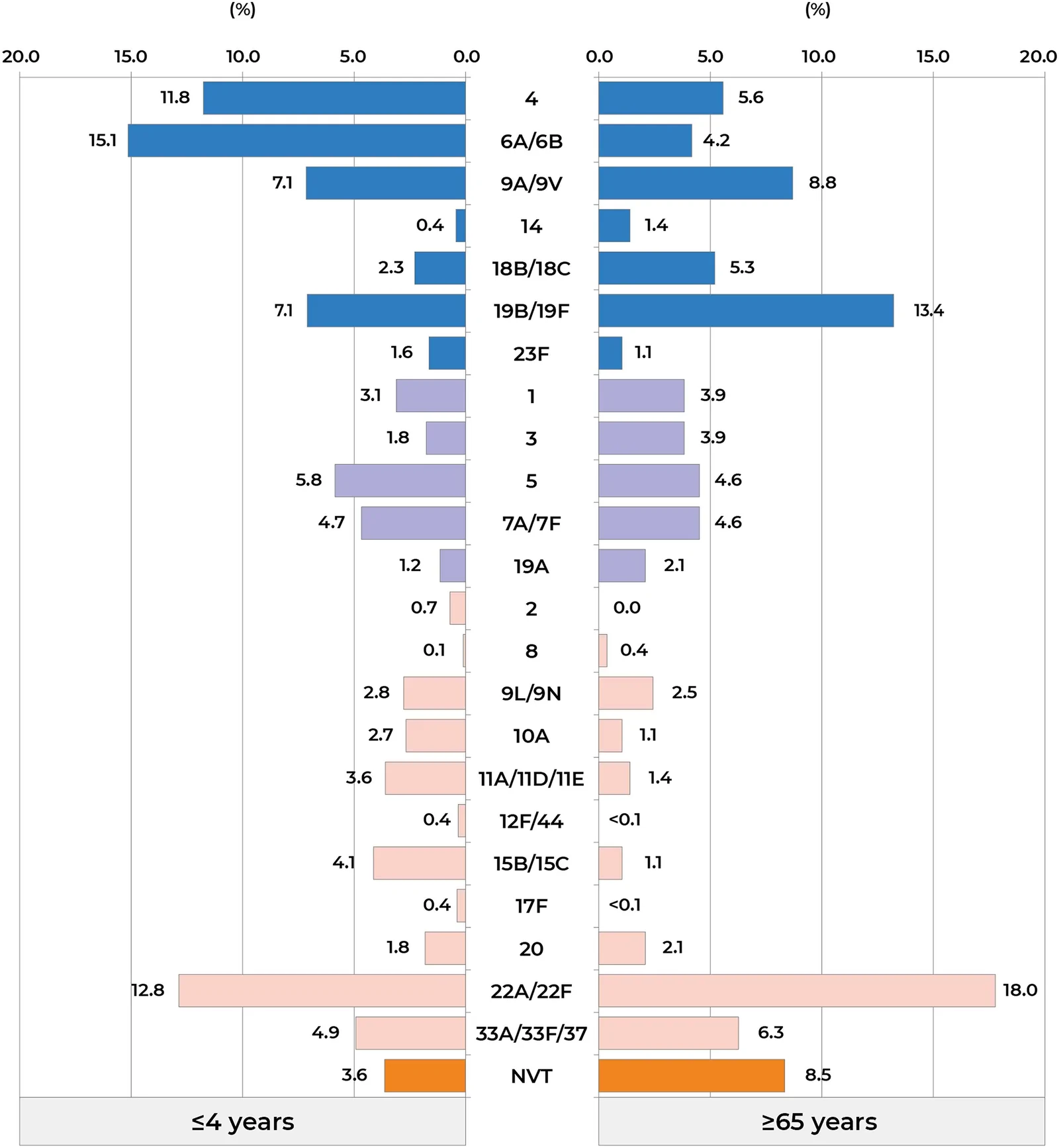
Relative frequencies of *Streptococcus pneumoniae* serotypes/serogroups among subjects aged ≤4 y and ≥65 y, according to PCV7, PCV13, or PPSV23. Pooled surveillance seasons. PCV7, 7-valent pneumococcal conjugate vaccine; PCV13, 13-valent pneumococcal conjugate vaccine; PPSV23, 23-valent pneumococcal polysaccharide vaccine.

Age-stratified analysis revealed marked differences (Figure 3). In children ≤4 years, the serotype profile resembled that of the full population but showed a predominance of 6A/6B. Logistic regression confirmed strong associations between carriage in younger age groups and several vaccine serotypes: PCV types 4 (OR & 3.5; 95% CI: 2.1–5.9), 5 (OR & 2.1; 95% CI: 1.1–3.7), and 6A/6B (OR & 7.7; 95% CI: 4.2–14.1), as well as PPSV23 serotypes 10A, 11A/11D/11E, and 15B/15C, although some estimates presented wide CIs. Conversely, NVTs were significantly associated with individuals aged ≥65 years.

Importantly, analysis restricted to the most recent surveillance years (2017–2020) confirmed the persistence of vaccine serotypes and ongoing community transmission, despite high pediatric vaccination coverage (data not shown).

**Figure 4.**
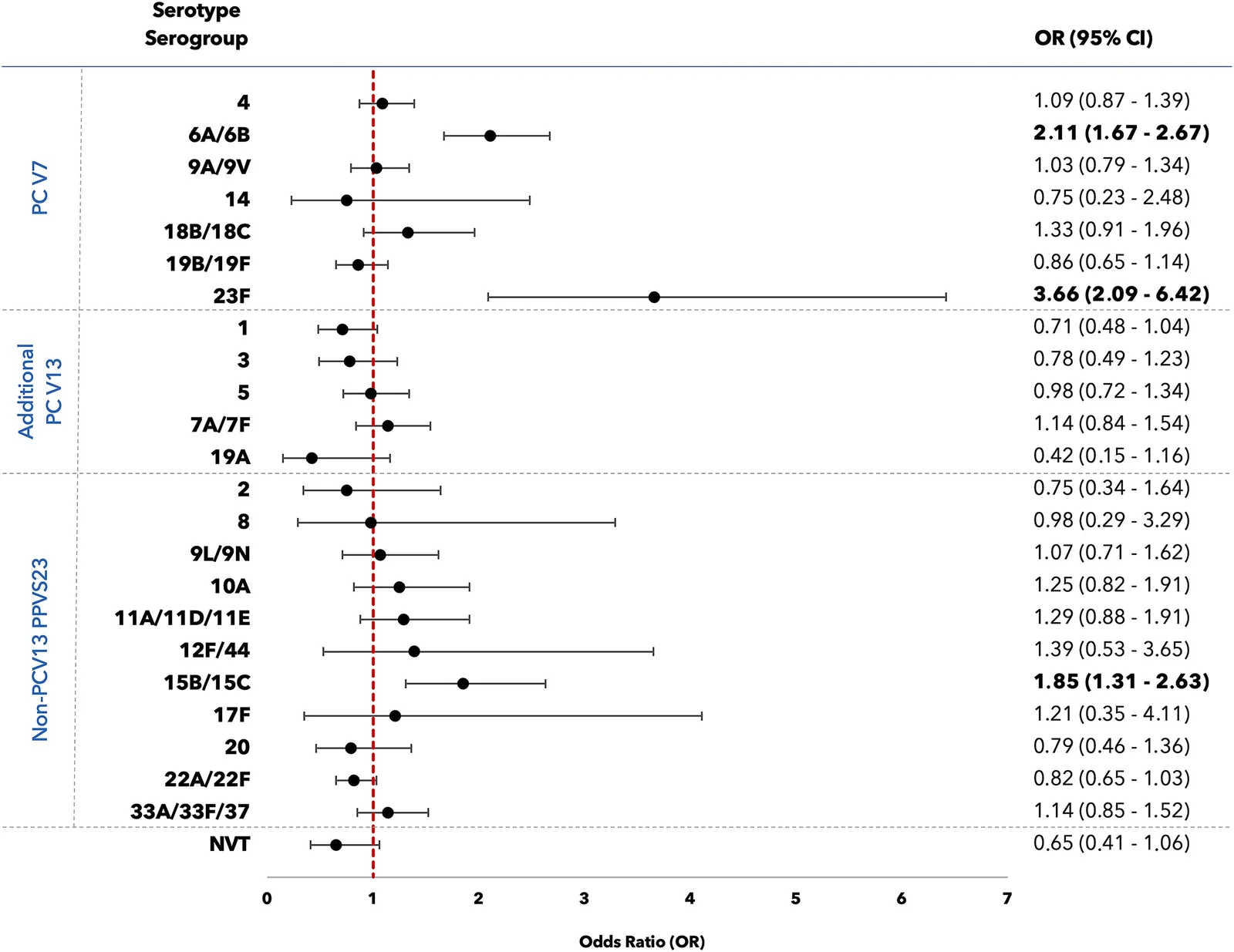
Forest plot showing the risk association between hRSV and pneumococcal serotype detection. hRSV-positive versus hRSV-negative (reference group: hRSV-negative). Pooled surveillance seasons. Statistically significant odds ratios are reported in bold (increased risk) and italic (decreased risk). NVTs, nonvaccine serotypes; hRSV, respiratory syncytial virus.

The distribution of colonizing serotypes was also compared based on viral coinfection. Influenza-positive subjects showed no substantial differences, except for serogroup 22A/22F, which was significantly associated with influenza infection, whereas 15B/15C showed a negative correlation (Supplementary Figure 4). Strong associations emerged between hRSV and serotypes 6A/6B (OR & 2.11; 95% CI: 1.67–2.67) and 23F (OR & 3.66; 95% CI: 2.09–6.43) and serogroup 15B/15C (OR & 1.86; 95% CI: 1.31–2.63) (Figure 4).

**Figure 5.**
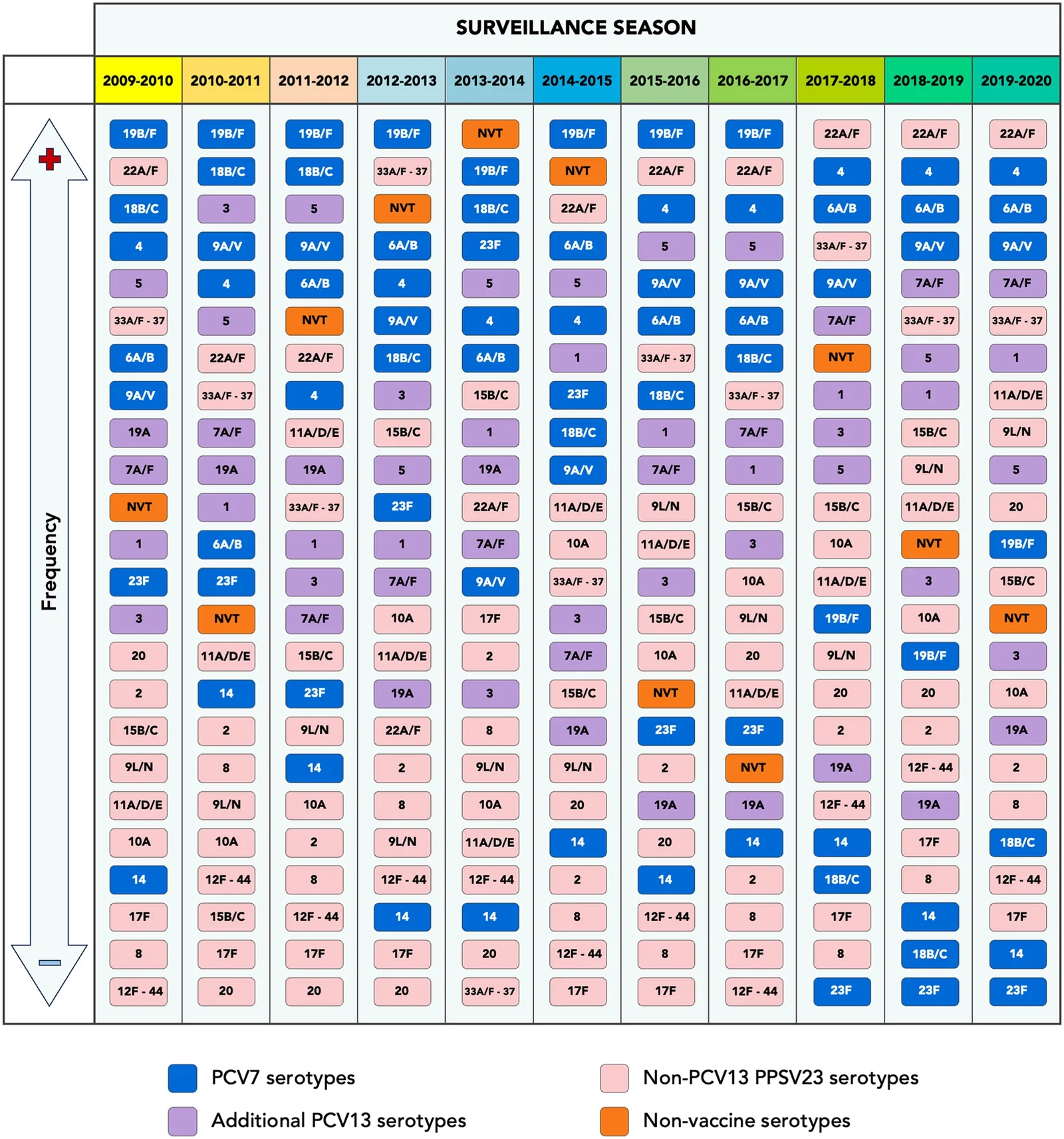
Seasonal fluctuation of pneumococcal serotypes included in PCV7, PCV13, or PPSV23 vaccines, according to each surveillance season. PCV7, 7-valent pneumococcal conjugate vaccine; PCV13, 13-valent pneumococcal conjugate vaccine; PPSV23, 23-valent pneumococcal polysaccharide vaccine.

Long-term dynamics of circulating serotypes are illustrated in Figure 5 and Supplementary Figures 5–8. Among PCV7 types, serogroup 19B/19F predominated until 2016–2017 before declining sharply, while serotype 4 consistently ranked among the top 10 and showed a steady increase, as did 6A/6B and 9A/9V. In contrast, serotypes 18B/18C and 19A, initially frequent, dramatically decreased over time. Serotype 23F peaked in 2014–2015 and then declined.

Finally, among those included in PPSV23, serogroups 22A/22F and 33A/33F/37 ranked within the highest position during the first 3 seasons monitored, although they circulated at low prevalences in the population. These serotypes progressively increased their frequencies over time, becoming 2 of the predominant serogroups, whereas 8, 9L/9N, 11A/11D/11E, 12F/44, and 20 showed the fastest increase in recent times.

## DISCUSSION

The present study aimed to explore impact and serotype-specific patterns of pneumococcal oropharyngeal carriage in Sicily (Italy). It offers valuable insight into the burden of oropharyngeal carriage of *S. pneumoniae* and the long-term dynamics of circulating serotypes among individuals of all ages. The findings span a decade following the introduction of the 13-valent pneumococcal conjugate vaccine as a universal childhood immunization and extend up to the beginning of the SARS-CoV-2 (severe acute respiratory syndrome coronavirus 2) pandemic. Several key outcomes emerged:

1. Pneumococcal colonization remained appreciable even among elderly individuals.
2. A marked decline in PCV serotypes (VTs) occurred following vaccine implementation, accompanied by a rise in NVTs.
3. Pneumococcal conjugate vaccine serotypes subsequently re-emerged several years later, despite long-term high vaccine coverage in children.
4. Serotype distribution showed strong age dependency.
5. Respiratory viruses, particularly hRSV, appeared to act as triggers for pneumococcal colonization.

Overall, pneumococcal carriage prevalence remained relatively stable throughout the study, except in periods when the median age of participants was higher, leading to a reduced proportion of carriers. The odds of carriage were up to 12 times greater in children aged 2–4 years compared with elderly individuals, corroborating observations from other research reporting the highest carriage rates among young children [24], particularly within the first 2 years of life in high-income countries [25]. While the inverse association between age and pneumococcal colonization is well documented [26], our findings emphasize that an average of at least 10% of adults and elderly individuals were colonized. This highlights an often underestimated reservoir of pneumococcus among older populations.

Carriage in adults is comparatively understudied, and existing reports are insufficient to establish a comprehensive understanding of adult colonization dynamics. Moreover, low prevalence estimates in older individuals have largely come from culture-based methods [27], which, despite being highly specific, may lack sensitivity for pneumococcal detection [28]. For instance, studies from the United States and England have estimated carriage in older adults at only 1.8%–2.2% [29, 30], while similar levels were observed in Portugal [31]. In contrast, a meta-analysis from Latin America [32] reported a pooled prevalence of 26% among elderly individuals, suggesting that adults may serve as an important pneumococcal reservoir in specific contexts, particularly where vaccine coverage differs [33]. These discrepancies underline the significance of local epidemiological and immunization conditions in shaping pneumococcal carriage trends.

Although young children remain the primary reservoir for pneumococcus and are the main source of transmission to older age groups, the presence of oropharyngeal colonization among elderly individuals warrants attention. Since colonization often precedes IPD, persistent carriage in older, vulnerable populations—who in many settings tend to be poorly vaccinated—raises considerable public health concerns [34].

As reported in multiple studies, our results demonstrate an initial post-PCV13 decline in colonization with VTs, accompanied by serotype replacement involving NVTs. This pattern has been observed globally following the uptake of PCVs, reflecting the impact of strong vaccine pressure on circulating serotypes. Portugal, for example, documented a drop in PCV13 serotype carriage from 47.6% prior to PCV13 introduction to just 10.7% by 2018–2020 [14]. Similar trends have been observed in England and Wales [35], Pakistan [15], the United States [36], and various European countries [37], where extensive childhood vaccination rapidly reshaped serotype ecology.

Despite the wealth of short-term data, fewer investigations have evaluated long-term serotype trends in regions with sustained high vaccine uptake [16, 33]. Our long-term analysis revealed, unexpectedly, a resurgence of some VTs in recent years. Although carriage levels varied by serotype and over time, certain PCV serotypes—such as 1, 4, 5, 6A/6B, 7A/7F, and 9A/9V—persisted in the population. While these serotypes currently do not represent a significant cause of IPD in infants in Sicily [8], their persistence in carriers is noteworthy. More importantly, serotypes 3, 14, 19A, 19B/19F, 8, and 10A demonstrated ongoing colonization and are associated with severe IPD risk [38]. Many of these serotypes have been implicated in vaccine breakthrough infections and continue to account for a substantial proportion of IPD cases in Italy, especially among young children and the elderly [8].

Similar phenomena have been observed elsewhere. In France [39], serotypes 3, 19A, and 19F were linked to IPD vaccine failures 6 years post-PCV13 introduction, while microbiome analyses confirmed the persistence of vaccine serotypes in the respiratory tract [38]. In rural Gambia [40], PCV13 serotypes plateaued after a decade of vaccination, with serotypes 3, 6A, and 19F persisting across age groups. In the United Kingdom [24], after sequential PCV7 and PCV13 introduction, a long-term decline in vaccine serotypes was documented, except for serotypes 3, 19A, and 19F, which continued to circulate into the 2020s, albeit at lower levels.

Age-related differences in serotype carriage were also prominent in our cohort. Children had higher odds of carrying serotypes 4, 6A/6B, and 5, as well as PPSV23-associated serotypes, such as 10A, 11A/11D/11E, and 15B/15C. Findings from other countries show similar trends but also underscore local variability. For example, in England [29], serotypes 10A, 11A, and 15B/15C predominated among children ≤4 years, whereas serotypes 4 and 6A/6B were more frequently detected in adults. In Gambia [16], serotype 19F persisted among children aged 0–4 years, while serotype 3 predominated in those aged 5–14 years. Israeli research further demonstrated different serotype patterns between children ≤2 years and those aged 2–5 years, with the latter group showing a distribution resembling that observed in adults with pneumococcal disease [41]. This may suggest waning postvaccination protection over time, especially among 5–9-year-olds, who in some studies showed higher odds of VT carriage than infants [16].

Because our surveillance enrolled individuals presenting with ILI, we also examined the effect of respiratory viruses as potential facilitators of pneumococcal colonization. Although carriage rates followed age patterns similar to those observed in healthy populations, viral codetection was strongly associated with pneumococcal carriage. This association was particularly marked for hRSV, which correlated with several clinically relevant serotypes, including PCV serotypes 6A/6B and 23F and PPSV23 serogroup 15B/15C.

Microbial interplay within the upper respiratory tract can be synergistic or competitive [42]. Several studies have shown that viral infections may enhance pneumococcal colonization density or facilitate acquisition either in older adults [43] or children [44]. Influenza virus can reshape the mucosal environment, suppress host immunity, and increase pneumococcal transmission among contacts [45]. These interactions have important implications for immunization strategies [46], particularly regarding live attenuated viral vaccines [47]. Respiratory syncytial virus has been demonstrated to directly increase pneumococcal adherence, proliferation, and virulence through altered immune responses [48]. Experimental studies suggest that hRSV infection increases susceptibility to secondary pneumococcal colonization and disease severity [49].

Some limitations of the study design should be taken into consideration. As the study was conducted within a single geographic region, the results may have limited generalizability and, not necessarily, represent the whole country. Nevertheless, because Sicily is one of the most populous regions of Italy and surveillance covered all provinces, we are confident in the representativeness of the results. A further limit concerns the lack of individual pneumococcal vaccination status, which did not allow for stratified analyses by immunization history. Moreover, sampling was limited to oropharyngeal specimens, and this may have biased the real impact of pneumococcal carriage, particularly in the pediatric population. Additionally, all participants were symptomatic patients presenting ILI, and this health condition may have further influenced the estimate of carriage prevalence. However, available evidence suggests that symptom-based sampling does not substantially distort the distribution of pneumococcal serotypes [50].

Despite these limitations, our work has several significant strengths. This investigation represents, to our knowledge, the first long-term analysis in Italy, which allowed evaluating pneumococcal oropharyngeal carriage and serotype trends across all ages over more than a decade of PCV use. Moreover, the molecular approach also enabled assessment of multiple colonizing serotypes, offering a broader representation of pneumococcal ecology than culture-based methods.

Ultimately, we found that while pneumococcal colonization remains highest among children, a considerable proportion of elderly individuals also harbor the pathogen. Respiratory viral coinfection appears to promote carriage, and persistent circulation of vaccine serotypes raises important concerns.

Collectively, these findings highlight the persistent circulation of vaccine serotypes, despite 2 decades of high PCV coverage in the pediatric population, and underscore the importance of ongoing surveillance and re-evaluation of immunization strategies, especially as pneumococcal serotype ecology continues to evolve. Monitoring carriage through community-based respiratory surveillance systems may be key to guiding future vaccine policy, assessing indirect protection, and anticipating shifts in pneumococcal disease burden.

## Supplementary Material

jiag150_Supplementary_Data

## Supplementary Data

Supplementary materials are available at *The Journal of Infectious Diseases* online (http://jid.oxfordjournals.org/). Supplementary materials consist of data provided by the author that are published to benefit the reader. The posted materials are not copyedited. The contents of all supplementary data are the sole responsibility of the authors. Questions or messages regarding errors should be addressed to the author.

## References

[1] Garcia Quesada M, Peterson ME, Bennett JC, et al Serotype distribution of remaining invasive pneumococcal disease after extensive use of ten-valent and 13-valent pneumococcal conjugate vaccines (the PSERENADE project): a global surveillance analysis. Lancet Infect Dis 2025; 25:445–56. 10.1016/S1473-3099(24)00588-7 PMC11947070 39706205

[2] Ditzel K, Giardina F, Ten Oever J, Cremers AJH. Risk conditions for invasive pneumococcal disease in adults: a systematic review and meta-analysis. EClinicalMedicine 2025; 89:103522. 10.1016/j.eclinm.2025.103522 PMC12550179 41140448

[3] Vojtek I, Buchy P, Doherty TM, Hoet B. Would immunization be the same without cross-reactivity? Vaccine 2019; 37:539–49. 10.1016/j.vaccine.2018.12.005 30591255

[4] Jang A-Y, Ji HJ, Kang EH, et al Cross-reactive immunity within pneumococcal serogroups: implications for next-generation vaccine development. Vaccine 2026; 73:128179. 10.1016/j.vaccine.2025.128179 41475154

[5] Feemster K, Hausdorff WP, Banniettis N, et al Implications of cross-reactivity and cross-protection for pneumococcal vaccine development. Vaccines 2024; 12:974. 10.3390/vaccines12090974 PMC11435891 39340006

[6] Flem E, Mouawad C, Palmu AA, et al Indirect protection in adults ≥18 years of age from pediatric pneumococcal vaccination: a review. Expert Rev Vaccines 2024; 23:997–1010. 10.1080/14760584.2024.2416229 39435466

[7] Hsiao A, Lewis N, Hansen J, et al Effectiveness of 13-valent pneumococcal conjugate vaccine against vaccine-type invasive pneumococcal disease in older adults. Vaccine 2025; 44:126543. 10.1016/j.vaccine.2024.126543 39637487

[8] Rapporto ISS Sorveglianza RIS-2/2024—Sorveglianza nazionale delle malattie batteriche invasive . Dati 2021–2023. Epicentro. 2024. Available at: https://www.iss.it/documents/20126/9840005/RIS+2-2024.pdf/67f460ad-111b-9fd9-24bd-4e23160ad864?t=1734614841773. Accessed 18 February 2026.

[9] European Centre for Disease Prevention and Control (ECDC) . Invasive pneumococcal disease—Annual Epidemiological Report for 2022. European Centre for Disease Prevention and Control (ECDC). 2022. Available at: https://www.ecdc.europa.eu/sites/default/files/documents/PNEU_AER_2022_Report.pdf. Accessed 18 February 2026.

[10] Navarro-Torné A, Montuori EA, Kossyvaki V, Méndez C. Burden of pneumococcal disease among adults in Southern Europe (Spain, Portugal, Italy, and Greece): a systematic review and meta-analysis. Hum Vaccin Immunother 2021; 17:3670–86. 10.1080/21645515.2021.1923348 PMC8437551 34106040

[11] Ministero della Salute—Repubblica Italiana . Vaccinazioni dell’età pediatrica e dell’adolescenza—Coperture vaccinali. Ministero della Salute—Repubblica Italiana. Available at: https://www.salute.gov.it/portale/documentazione/p6_2_8_1_1.jsp?lingua=italiano&id=38. Accessed 18 February 2026.

[12] Di Valerio Z, La Fauci G, Scognamiglio F, et al Pneumococcal vaccine uptake among high-risk adults and children in Italy: results from the OBVIOUS project survey. BMC Public Health 2024; 24:736. 10.1186/s12889-024-18216-3 PMC10921627 38454392

[13] Trimble A, Connor V, Robinson RE, et al Pneumococcal colonisation is an asymptomatic event in healthy adults using an experimental human colonisation model. PLoS One 2020; 15:e0229558. 10.1371/journal.pone.0229558 PMC7064211 32155176

[14] Candeias C, Almeida ST, Paulo AC, et al Streptococcus pneumoniae carriage, serotypes, genotypes, and antimicrobial resistance trends among children in Portugal, after introduction of PCV13 in national immunization program: a cross-sectional study. Vaccine 2024; 42:126219. 10.1016/j.vaccine.2024.126219 39146858

[15] Iqbal I, Shahid S, Kanwar S, et al Pneumococcal carriage and changes in serotype distribution post- PCV13 introduction in children in Matiari, Pakistan. Vaccine 2024; 42:126238. 10.1016/j.vaccine.2024.126238 PMC11413484 39168078

[16] Osei I, Mendy E, van Zandvoort K, et al Effect of the 13-valent pneumococcal conjugate vaccine on pneumococcal carriage in rural Gambia 10 years after its introduction: a population-based cross-sectional study. Vaccine 2025; 56:127181. 10.1016/j.vaccine.2025.127181 40300436

[17] Wulffraat MT, van de Weijer NG, Wijmenga-Monsuur AJ, et al Evolving pneumococcal epidemiology and vaccine impact in The Netherlands, 2004–2024: carriage and invasive disease. J Infect 2025; 91:106658. 10.1016/j.jinf.2025.106658 41241235

[18] Wyllie AL, Rümke LW, Arp K, et al Molecular surveillance on Streptococcus pneumoniae carriage in non-elderly adults; little evidence for pneumococcal circulation independent from the reservoir in children. Sci Rep 2016; 6:34888. 10.1038/srep34888 PMC5054371 27713565

[19] Cleary DW, Jones J, Gladstone RA, et al Changes in serotype prevalence of Streptococcus pneumoniae in Southampton, UK between 2006 and 2018. Sci Rep 2022; 12:13332. 10.1038/s41598-022-17600-6 PMC9349173 35922536

[20] Istituto Superiore di Sanità (ISS) . Protocollo Operativo RespiVirNet 2024–2025. Epicentro. 2024. Available at: https://www.epicentro.iss.it/influenza/pdf/Protocollo%20Operativo%20RespiVirNet%20Stagione%202024-2025.pdf. Accessed 18 February 2026.

[21] Tramuto F, Maida CM, Randazzo G, et al Whole-genome sequencing and genetic diversity of human respiratory syncytial virus in patients with influenza-like illness in Sicily (Italy) from 2017 to 2023. Viruses 2024; 16:851. 10.3390/v16060851 PMC11209242 38932144

[22] Tramuto F, Maida CM, Randazzo G, et al Insights into genetic and antigenic characteristics of influenza A(H1N1)pdm09 viruses circulating in Sicily during the surveillance season 2023–2024: the potential effect on the seasonal vaccine effectiveness. Viruses 2024; 16:1644. 10.3390/v16101644 PMC11512306 39459976

[23] Velusamy S, Tran T, Mongkolrattanothai T, Walker H, McGee L, Beall B. Expanded sequential quadriplex real-time polymerase chain reaction (PCR) for identifying pneumococcal serotypes, penicillin susceptibility, and resistance markers. Diagn Microbiol Infect Dis 2020; 97:115037. 10.1016/j.diagmicrobio.2020.115037 32265073

[24] Tiley KS, Ratcliffe H, Voysey M, et al Nasopharyngeal carriage of pneumococcus in children in England up to 10 years after 13-valent pneumococcal conjugate vaccine introduction: persistence of serotypes 3 and 19A and emergence of 7C. J Infect Dis 2023; 227:610–21. 10.1093/infdis/jiac376 PMC9978316 36130327

[25] Simell B, Auranen K, Käyhty H, et al The fundamental link between pneumococcal carriage and disease. Expert Rev Vaccines 2012; 11:841–55. 10.1586/erv.12.53 22913260

[26] Le Polain de Waroux O, Flasche S, Prieto-Merino D, Edmunds WJ. Age-dependent prevalence of nasopharyngeal carriage of streptococcus pneumoniae before conjugate vaccine introduction: a prediction model based on a meta-analysis. PLoS One 2014; 9:e86136. 10.1371/journal.pone.0086136 PMC3900487 24465920

[27] Flamaing J, Peetermans WE, Vandeven J, Verhaegen J. Pneumococcal colonization in older persons in a nonoutbreak setting. J Am Geriatr Soc 2010; 58:396–8. 10.1111/j.1532-5415.2009.02700.x 20370873

[28] Miellet WR, Almeida ST, Trzciński K, Sá-Leão R. Streptococcus pneumoniae carriage studies in adults: importance, challenges, and key issues to consider when using quantitative PCR-based approaches. Front Microbiol 2023; 14:1122276. 10.3389/fmicb.2023.1122276 PMC9994646 36910231

[29] El Safadi D, Hitchins L, Howard A, et al Pneumococcal carriage and disease in adults in England, 2011–2019: the importance of adults as a reservoir for pneumococcus in communities. J Infect Dis 2025; 231:e17–27. 10.1093/infdis/jiae351 PMC11793058 39013016

[30] Milucky J, Carvalho MG, Rouphael N, et al Streptococcus pneumoniae colonization after introduction of 13-valent pneumococcal conjugate vaccine for US adults 65 years of age and older, 2015–2016. Vaccine 2019; 37:1094–100. 10.1016/j.vaccine.2018.12.075 PMC6371770 30685247

[31] Almeida ST, Nunes S, Santos Paulo AC, et al Low prevalence of pneumococcal carriage and high serotype and genotype diversity among adults over 60 years of age living in Portugal. PLoS One 2014; 9:e90974. 10.1371/journal.pone.0090974 PMC3946249 24604030

[32] Brizuela M, Palermo MC, Alconada T, et al Nasopharyngeal carriage of Streptococcus pneumoniae in Latin America and the Caribbean: a systematic review and meta-analysis. PLoS One 2024; 19:e0297767. 10.1371/journal.pone.0297767 PMC11104613 38768099

[33] Davis SM, Deloria-Knoll M, Kassa HT, O’Brien KL. Impact of pneumococcal conjugate vaccines on nasopharyngeal carriage and invasive disease among unvaccinated people: review of evidence on indirect effects. Vaccine 2013; 32:133–45. 10.1016/j.vaccine.2013.05.005 23684824

[34] Perniciaro S, van der Linden M. Pneumococcal vaccine uptake and vaccine effectiveness in older adults with invasive pneumococcal disease in Germany: a retrospective cohort study. Lancet Reg Health Eur 2021; 7:100126. 10.1016/j.lanepe.2021.100126 PMC8454757 34557837

[35] Devine VT, Cleary DW, Jefferies JMC, et al The rise and fall of pneumococcal serotypes carried in the PCV era. Vaccine 2017; 35:1293–8. 10.1016/j.vaccine.2017.01.035 28161425

[36] Weinberger DM, Malley R, Lipsitch M. Serotype replacement in disease after pneumococcal vaccination. Lancet 2011; 378:1962–73. 10.1016/S0140-6736(10)62225-8 PMC3256741 21492929

[37] Allemann A, Frey PM, Brugger SD, Hilty M. Pneumococcal carriage and serotype variation before and after introduction of pneumococcal conjugate vaccines in patients with acute otitis media in Switzerland. Vaccine 2017; 35:1946–53. 10.1016/j.vaccine.2017.02.010 28279564

[38] Yildirim M, Keskinocak P, Hinderstein S, et al A comprehensive analysis of serotype-specific invasive capacity, clinical presentations, and mortality trends of invasive pneumococcal disease. Vaccine 2025; 47:126692. 10.1016/j.vaccine.2024.126692 PMC12859698 39778476

[39] Levy C, Varon E, Ouldali N, Béchet S, Bonacorsi S, Cohen R. Changes in invasive pneumococcal disease spectrum after 13-valent pneumococcal conjugate vaccine implementation. Clin Infect Dis 2020; 70:446–54. 10.1093/cid/ciz221 30869777

[40] Usuf E, Bottomley C, Gladstone R, et al Persistent and emerging pneumococcal carriage serotypes in a rural Gambian community after 10 years of pneumococcal conjugate vaccine pressure. Clin Infect Dis 2021; 73:e3825–35. 10.1093/cid/ciaa856 32584973

[41] Wyllie AL, Warren JL, Regev-Yochay G, Givon-Lavi N, Dagan R, Weinberger DM. Serotype patterns of pneumococcal disease in adults are correlated with carriage patterns in older children. Clin Infect Dis 2021; 72:e768–75. 10.1093/cid/ciaa1480 PMC8315131 32989457

[42] Paulo AC, Lança J, Almeida ST, Hilty M, Sá-Leão R. The upper respiratory tract microbiota of healthy adults is affected by Streptococcus pneumoniae carriage, smoking habits, and contact with children. Microbiome 2023; 11:199. 10.1186/s40168-023-01640-9 PMC10474643 37658443

[43] Miellet WR, van Veldhuizen J, Nicolaie MA, et al Influenza-like illness exacerbates pneumococcal carriage in older adults. Clin Infect Dis 2021; 73:e2680–9. 10.1093/cid/ciaa1551 33124669

[44] Nyazika TK, Law A, Swarthout TD, et al Influenza-like illness is associated with high pneumococcal carriage density in Malawian children. J Infect 2020; 81:549–56. 10.1016/j.jinf.2020.06.079 PMC7375306 32711042

[45] Bosch AATM, Biesbroek G, Trzcinski K, Sanders EAM, Bogaert D. Viral and bacterial interactions in the upper respiratory tract. PLoS Pathog 2013; 9:e1003057. 10.1371/journal.ppat.1003057 PMC3542149 23326226

[46] Manna S, McAuley J, Jacobson J, et al Synergism and antagonism of bacterial-viral coinfection in the upper respiratory tract. mSphere 2022; 7:e0098421. 10.1128/msphere.00984-21 PMC8769199 35044807

[47] Peno C, Armitage EP, Clerc M, et al The effect of live attenuated influenza vaccine on pneumococcal colonisation densities among children aged 24–59 months in the Gambia: a phase 4, open label, randomised, controlled trial. Lancet Microbe 2021; 2:e656–65. 10.1016/S2666-5247(21)00179-8 PMC8632704 34881370

[48] Kirsebom FCM, Kausar F, Nuriev R, Makris S, Johansson C. Neutrophil recruitment and activation are differentially dependent on MyD88/TRIF and MAVS signaling during RSV infection. Mucosal Immunol 2019; 12:1244–55. 10.1038/s41385-019-0190-0 PMC6778055 31358860

[49] Mitsi E, Nikolaou E, Goncalves A, et al RSV and rhinovirus increase pneumococcal carriage acquisition and density, whereas nasal inflammation is associated with bacterial shedding. Cell Host Microbe 2024; 32:1608–20.e4. 10.1016/j.chom.2024.07.024 39181126

[50] Krone CL, Wyllie AL, van Beek J, et al Carriage of Streptococcus pneumoniae in aged adults with influenza-like-illness. PLoS One 2015; 10:e0119875. 10.1371/journal.pone.0119875 PMC4366201 25789854

