# Supplementary material for: Indirect Effect of Pneumococcal Conjugate Vaccines on Pneumococcal Colonization: Persistence and Dynamics of Vaccine Serotypes in Sicily (Italy) 11 Years Postintroduction, 2009–2020
Source: J Infect Dis. 2026 Mar 7;234(3):e690–701. doi: 10.1093/infdis/jiag150 (PMC13600418; doi:10.1093/infdis/jiag150)
Supplement: Supplementary file 1 [file jiag150_supplementary_data.pdf]

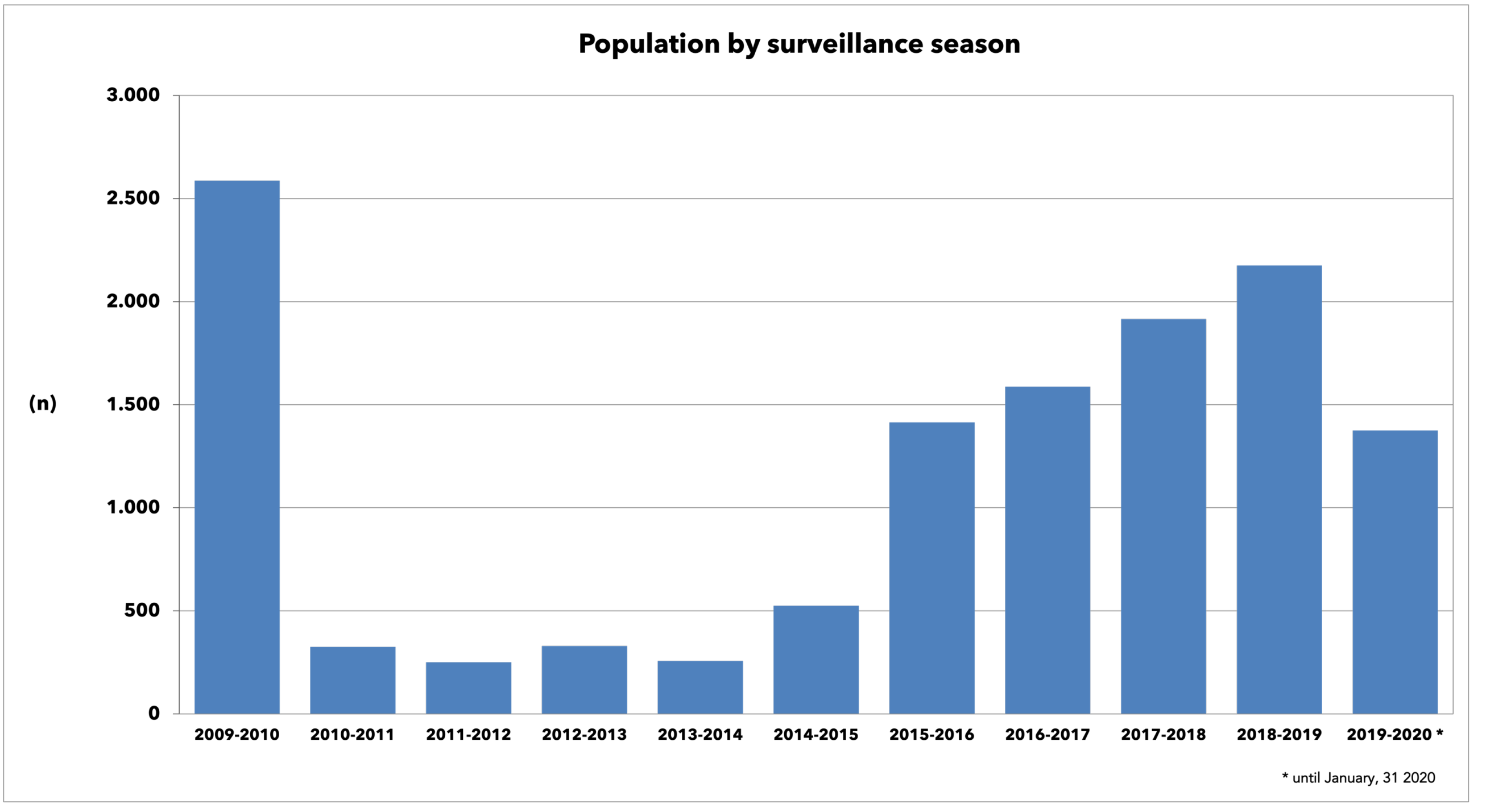

Supplementary Figure 1. Population sampled according to surveillance season.

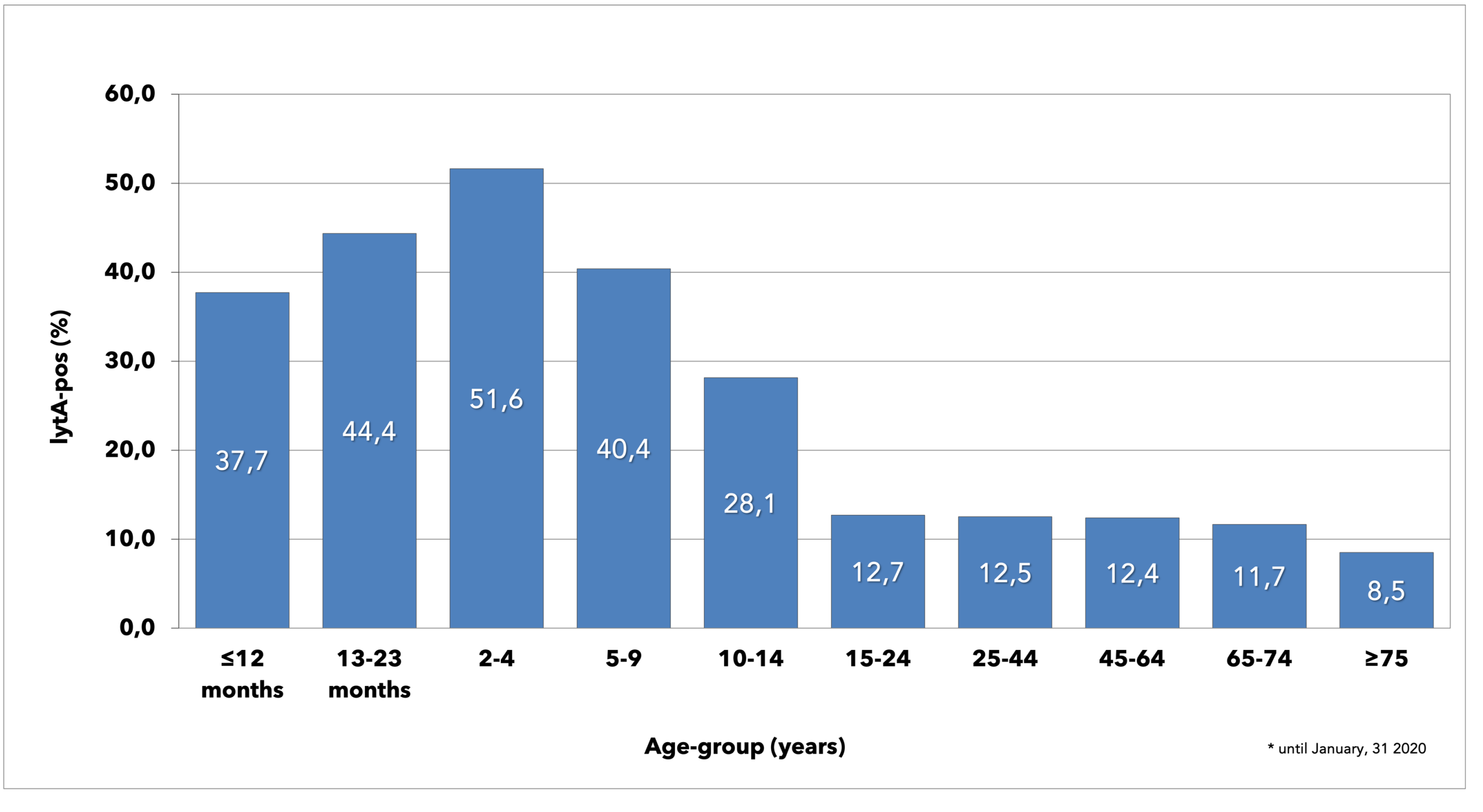

Supplementary Figure 2. Prevalence of pneumococcal carriage according to age-group. Pooled surveillance seasons.

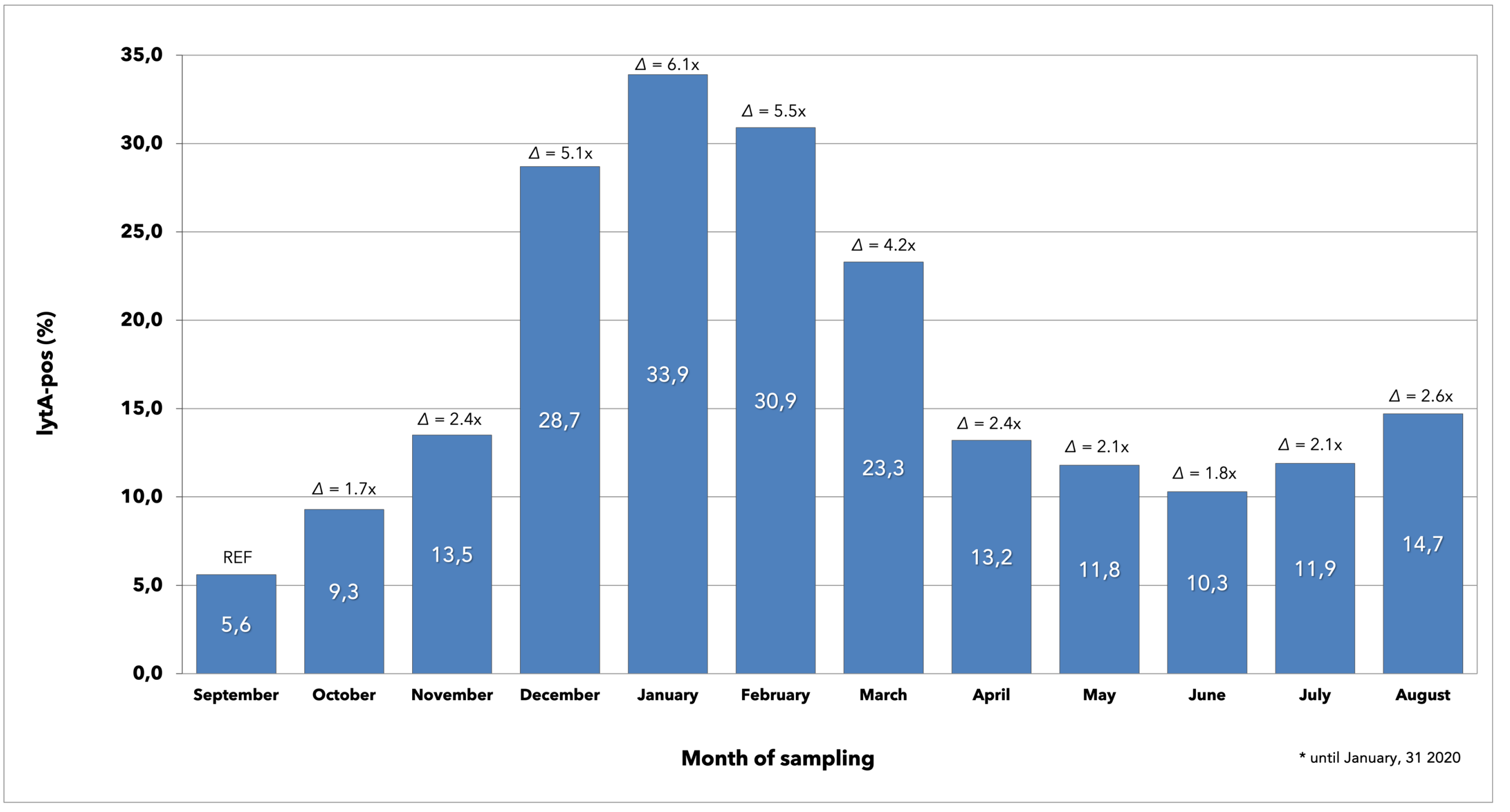

Supplementary Figure 3. Prevalence of pneumococcal carriage stratified by month of sampling. Pooled surveillance seasons.

Months are ordered in accordance with the seasonal period of surveillance. Δ represents the prevalence ratio, calculated by taking September as the reference month.

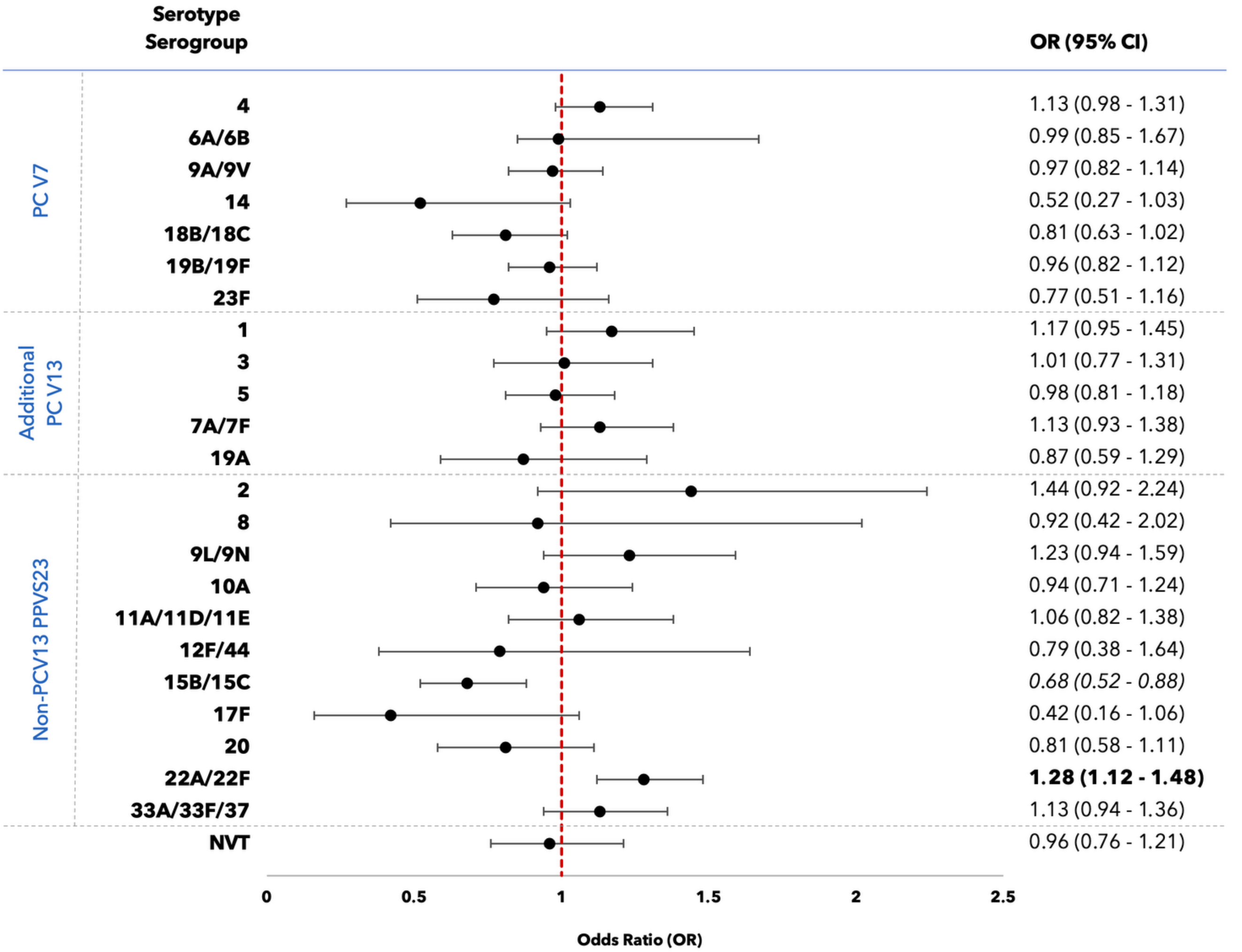

Supplementary Figure 4. Forest plot showing the risk association between influenza virus and pneumococcal serotype detection. Influenza-positive *vs.* influenza-negative (reference group: influenza-negative). Pooled surveillance seasons. Statistically significant odds ratios are reported in bold (increased risk) and italic (decreased risk). NVT=Non-vaccine serotypes.

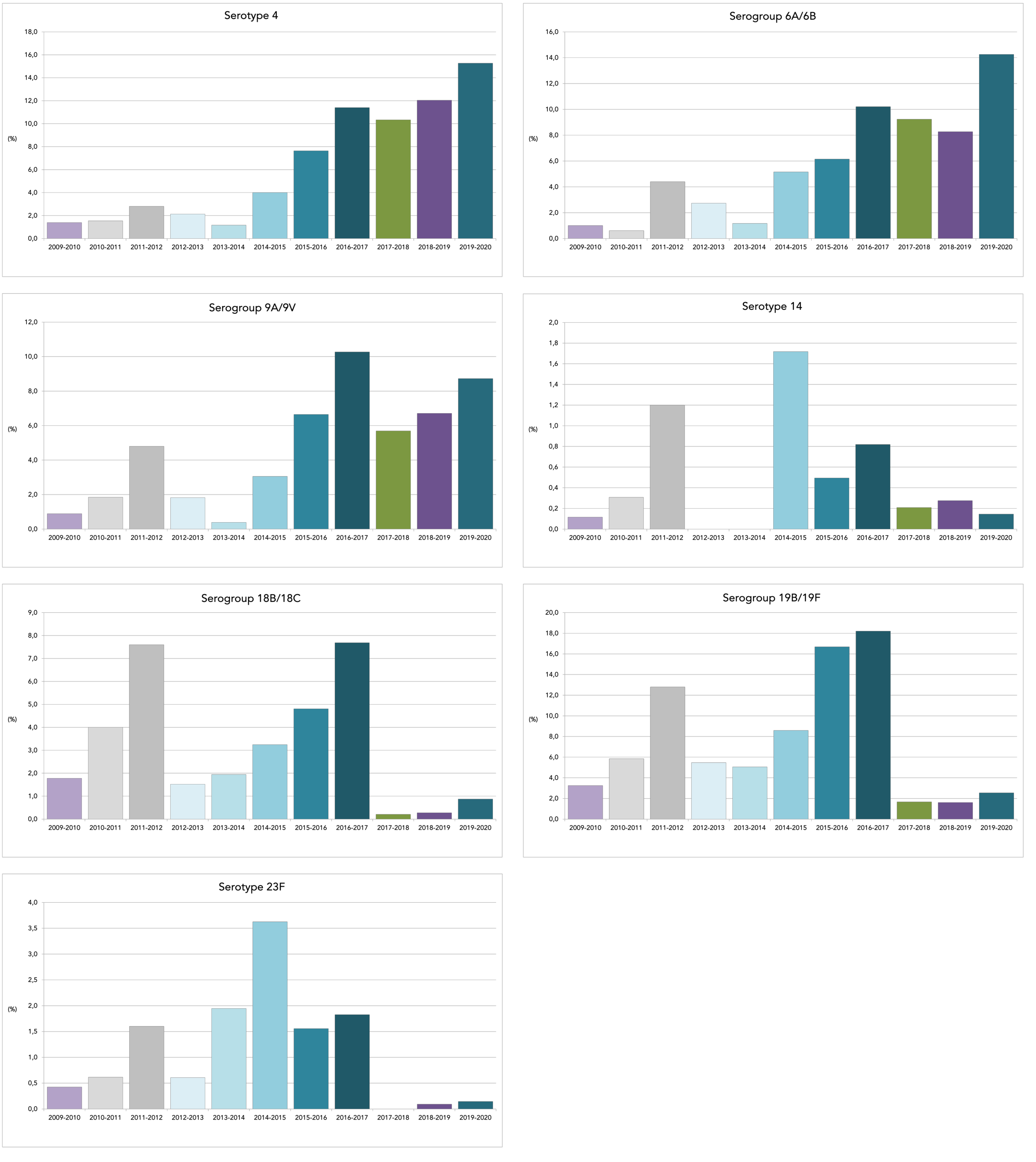

Supplementary Figure 5. Prevalence of *S. pneumoniae* serotypes / serogroups included in PCV7, according to each surveillance season.

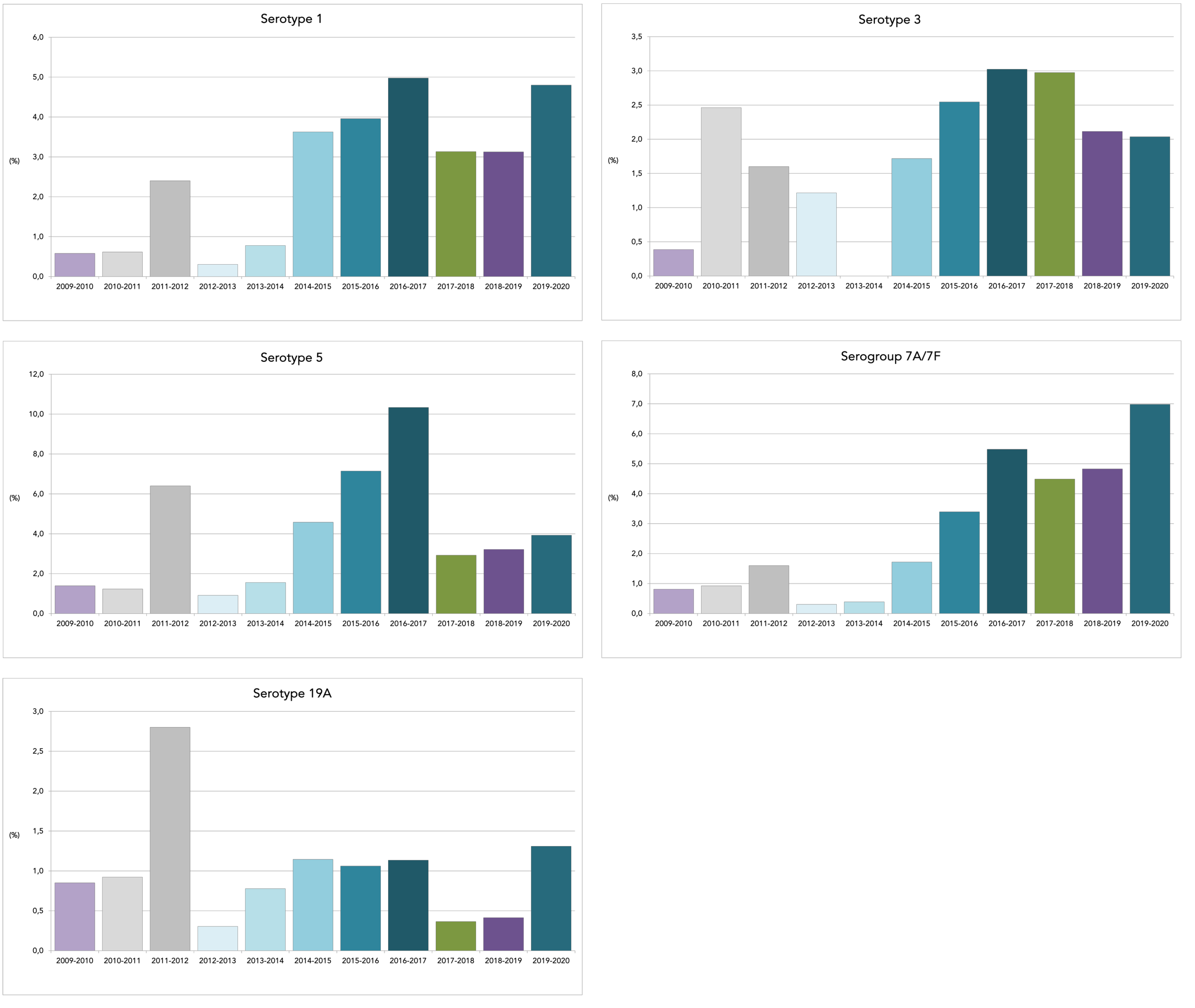

Supplementary Figure 6. Prevalence of *S. pneumoniae* serotypes / serogroups included in PCV13 (in addition to PCV7), according to each surveillance season.

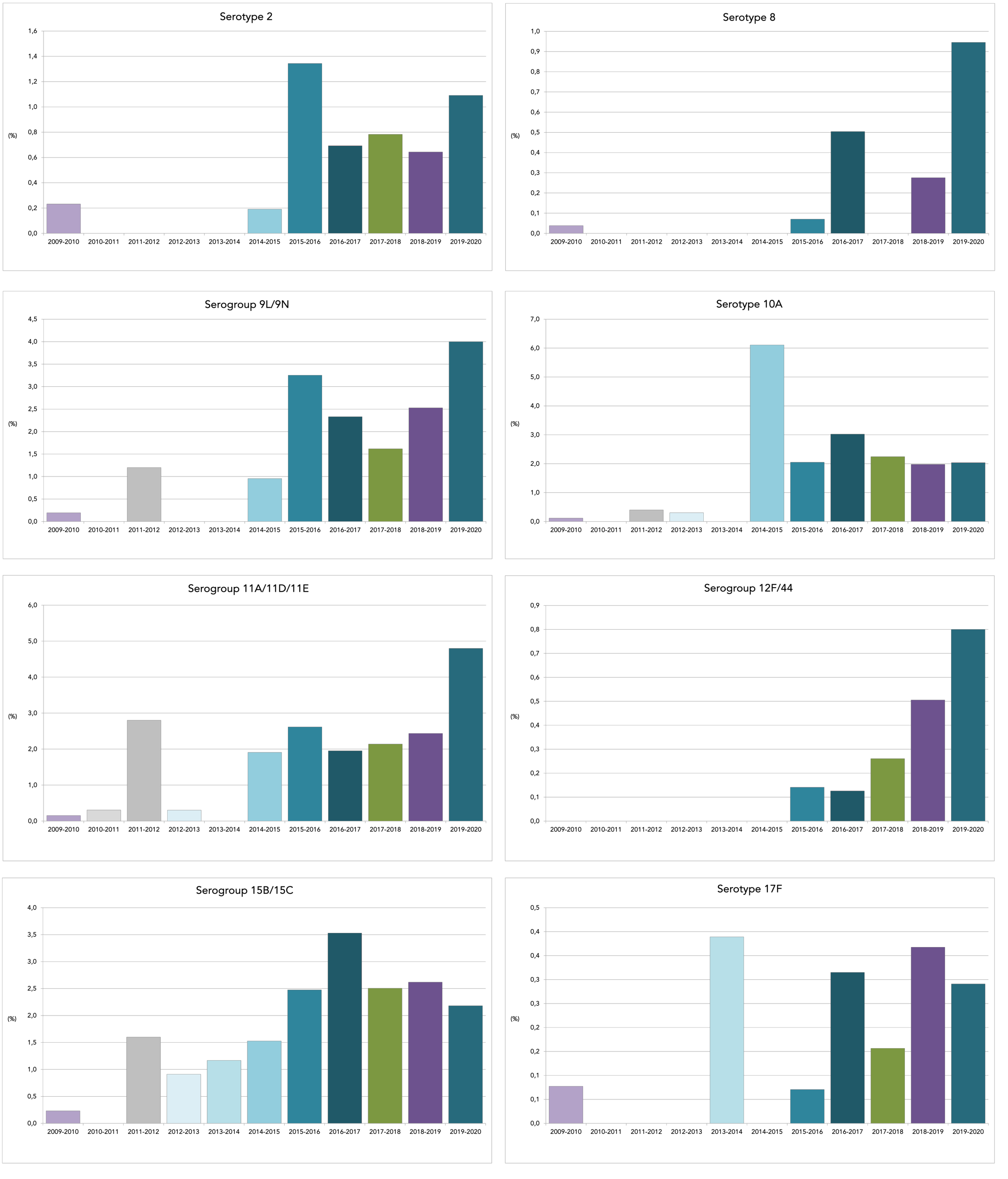

Supplementary Figure 7. (a) Prevalence of *S. pneumoniae* serotypes / serogroups included in PPPV23 (in addition to PCV7+PCV13), according to each surveillance season.

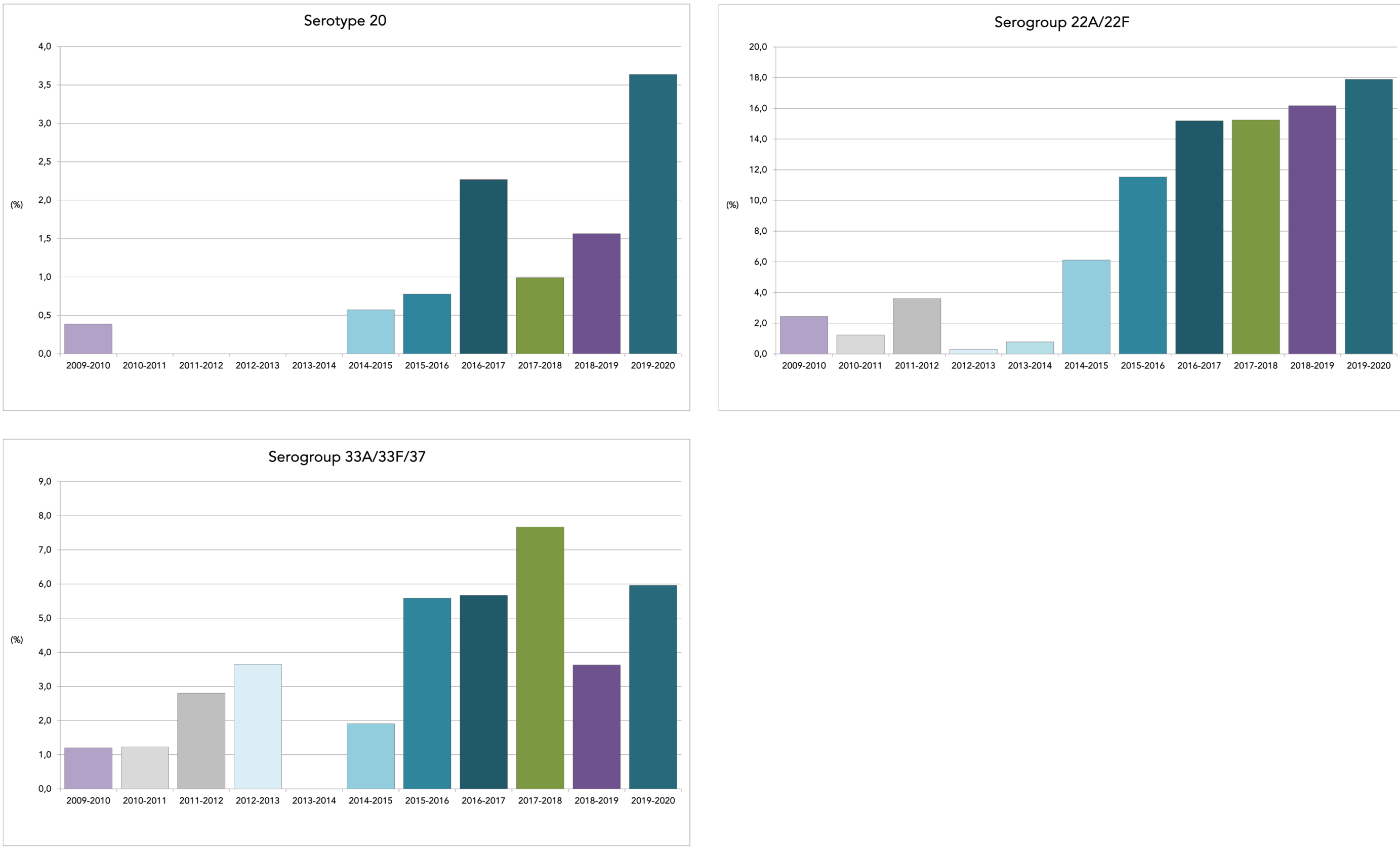

Supplementary Figure 7. (b) Prevalence of *S. pneumoniae* serotypes / serogroups included in PPPV23 (in addition to PCV7+PCV13), according to each surveillance season.

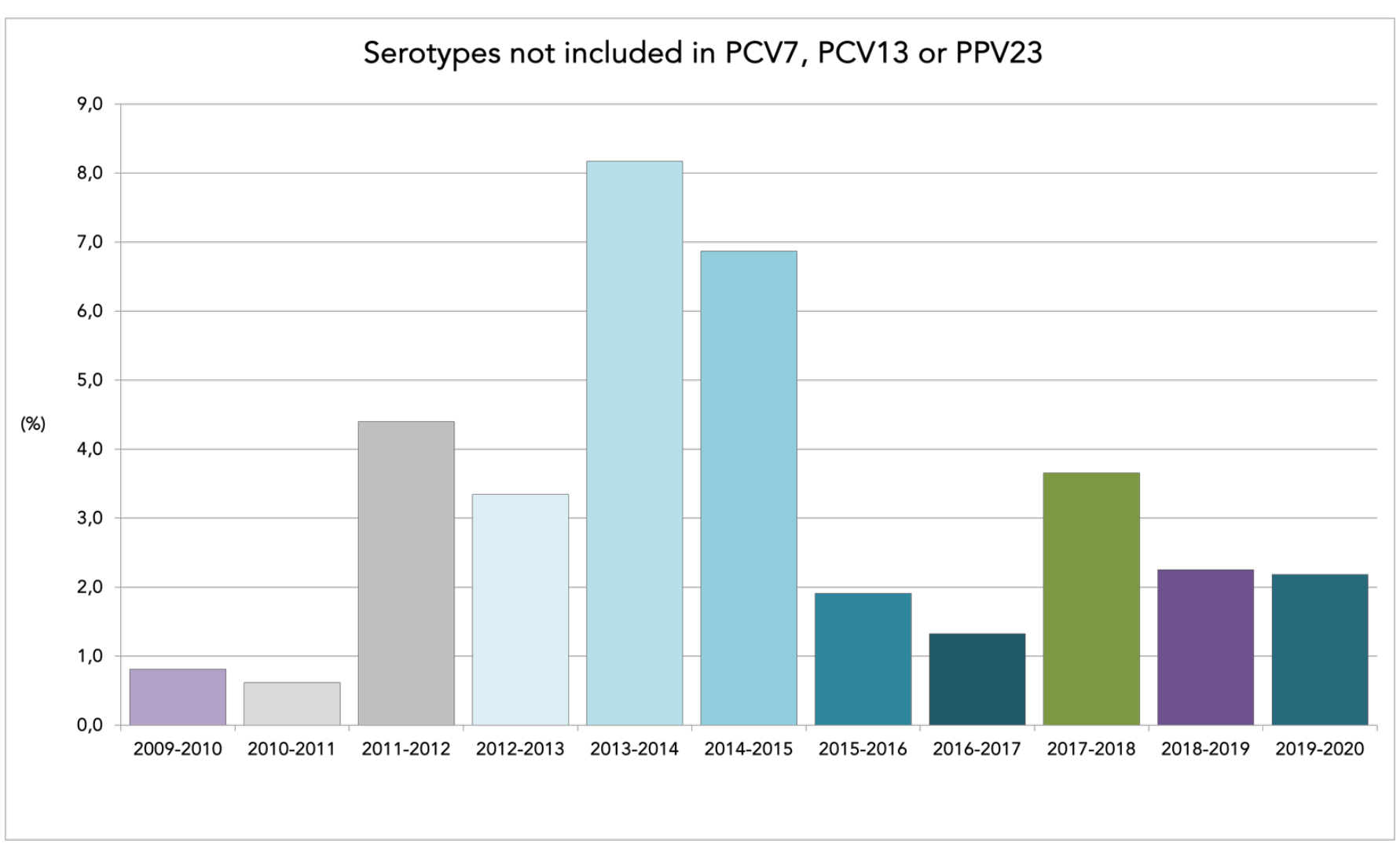

Supplementary Figure 8. Prevalence of *S. pneumoniae* serotypes / serogroups not included in PCV7, PCV13 and PPPV23, according to each surveillance season.
